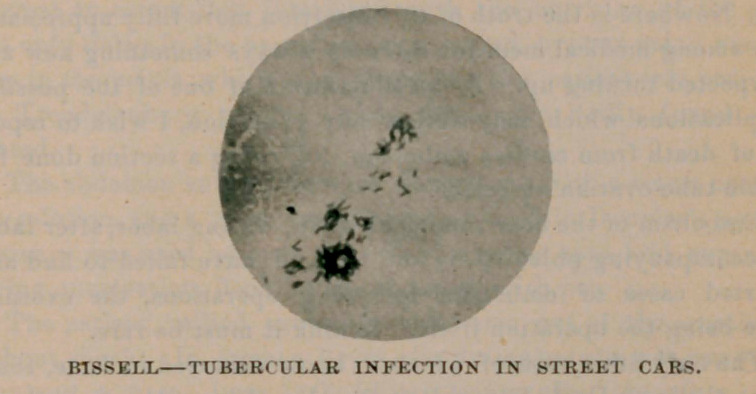# Tubercular Infection in Street Cars

**Published:** 1895-08

**Authors:** William G. Bissell

**Affiliations:** Bacteriologist, Department of Health, Buffalo, N. Y.


### TUBERCULAR INFECTION IN STREET CARS.

BY WILLIAM G. BISSELL, M. D.,
Bacteriologist, Department of Health, Buffalo, N. Y.

A SHORT time ago the Buffalo Street Railway Company adopted a rule looking to the prevention of expectoration on the floors of street cars. There was placed in each car a sign reading to the effect that "spitting on the floor of this car is positively prohibited." The result of the display of these signs was the lessening, to a small degree, the amount of expectoration on the floors of the cars.

The move was one in the right direction, and should be highly commended; but company rules of this nature are difficult to enforce without stronger legislation back of them, and some measure should be adopted to aid the company in its effort toward better street car sanitation.

During the past few years much has been stated as to the possible spreading of tubercular infection by careless and indiscriminate expectoration. Where is there a more common place for the spreading of such infection than the floors of street cars?

The sputum becomes dried, mixed with dust and easily disseminated by currents of air. During the year 1894, in the city of Buffalo, over 42,500,000 passengers were carried, in some 2,700 cars, by the street railway company, and one can appreciate by this number the very considerable amount of dried expectoration that must necessarily have been inhaled.

With a few exceptions, very little work has been done to demonstrate practically the possibility of street car sputum infection, calculations having been based on the fact that tubercle bacillus is usually present in the sputum of consumptives, and, undoubtedly, among the number that ride on the street cars annually there are several hundred persons suffering from the disease.

This point has been demonstrated to be a fact, for during the past three months fifty-six microscopical examinations have been made of selected samples from the floors of cars at the foot of

Main street, and four of these examinations revealed presence of tubercle bacilli.

I draw attention to one mount, of which I furnish a micro-photograph. This photograph shows a clump of tubercular organisms numbering in the hundreds, the sample having been collected from a Cold Spring car.

BISSELL—TUBERCULAR INFECTION IN STREET CARS.

The question now arises, How can the contaminating of cars by tubercular sputum be prevented? Principally in two ways:

First, by educating the public in general as to the danger of careless and indiscriminate expectoration, which step is being thoroughly taken at present by the department of health.

Second, by the passing of a city ordinance prohibiting the expectorating on the floors of cars, public buildings and similar places.

Until such ordinance has been passed, a rule of the street railway company, or of any private corporation, cannot be satisfactorily enforced. The point I wish to bring out in this short article is the necessity of such an ordinance, and the hope that legislation will be brought to bear on this point in the near future.

I am indebted to my colleague, Dr. Carpenter, for his assistance in the work of street car sputum contamination, and also to Dr. Herbert M. Hill for the micro-photograph above displayed.

10 ORTON PLACE.

---

ULYPTOL belongs in the same category with steresol. It is occasionally mentioned as a "new antiseptic." It was originally named and introduced in 1886, and is prepared by mixing six parts salicylic acid, one part carbolic acid and one part oil eucalyptus. It is also known as eulyptol, and the mixture is of service in treating wounds.—*American Therapist.*

## Figures and Tables

**Figure f1:**